# Discontinuation rate of Implanon and its associated factors among women who ever used Implanon in Dale District, Southern Ethiopia

**DOI:** 10.1186/s12905-018-0678-x

**Published:** 2018-11-20

**Authors:** Abreham Nageso, Achamyelesh Gebretsadik

**Affiliations:** School of Public and Environmental Health, Hawassa University, Hawassa, Ethiopia

**Keywords:** Implanon, Early discontinuation, Dale, Ethiopia

## Abstract

**Background:**

Early discontinuation of the Implanon contraceptive method and reasons for such discontinuation remains a major concern for family planning programs. In less developed countries, contraceptive discontinuation due to health concerns is generally higher, these complaints are often related to service quality. Significant numbers of women become exposed to conception after discontinuation and accidental pregnancies that end up with abortion & stillbirth. The aim of this study was to assess the early discontinuation rate of Implanon and identify its associated factors among women who ever used Implanon in 2016 in Dale district, Southern Ethiopia.

**Methods:**

Community based cross-sectional study design was conducted from January to February, 2017. A total number of 711 women who ever used Implanon in 2016 were selected using multistage sampling. The data were entered and cleaned in Epi Info and analyzed using SPSS. Multivariate logistic regression analysis was used to determine the effect of factors on the outcome variables. Finally, the results were presented using adjusted odds ratio (AOR) & confidence interval of 95%.

**Result:**

Early Implanon discontinuation rate in this study was 160 (23.4%) with a mean duration of Implanon use of 9.6 ± 2.5 months. The main reasons for discontinuation of Implanon were 55 (34.4%) the facing of side effects. Factors for discontinuation of Implanon were women age 20–24 years (AOR =. 44 (95% CI: 23-. 85), 25–29 years (AOR =. 52 (95% CI: 27-. 96), 35+ years, (AOR =. 08 (95% CI: 02-. 41), less likely to discontinue. Women who weren’t counseled about the side effects during Implanon insertion were 1.93 times (AOR = 1.93 (95% CI: 1.27–2.93), women who didn’t satisfied by the service (AOR = 2.55(CI: 95%: 1.63–3.97), women who didn’t appointed for follow up (AOR = 3.13 (CI: 95%: 2.0–4.95), women who didn’t choose the method by themselves (AOR = 1.83 (CI: 95%: 1.18–2.83) and women who didn’t have information on family planning before Implanon insertion (AOR = 1.52 (CI: 95%: 1.1–2.28) were the predictors of Implanon discontinuation.

**Conclusions:**

Implanon discontinuation rate in this study area was high. Appropriate counseling prior to insertion and proper follow up, autonomous choice will improve the continuation rate of Implanon.

## Background

Implanon is long acting and reversible sub dermal contraceptive. Implanon inhibits ovulation within 1 day of insertion and provides effective contraception for up to 3 years [1]. Once the women are inserted, they require little user compliance and it is with a prompt return to fertility immediately after removal [2]. Though it has a good feature, the utilization rate is still low among women in reproductive age around the globe [3].

Evidence strongly suggests that the provision of quality family planning services can increase uptake, prevalence, and decreases discontinuation of contraception [4]. Although many women who use Implanon contraceptive are happy with their choices, a significant number of women choose the method and then request early removal [5].

Use of less effective methods, infrequent use, and method discontinuation have an effect on the rate of unintended pregnancies. In different countries significant numbers of women become exposed to the risk of conception after discontinuation and accidental pregnancies that end up in miscarriage, stillbirth or abortion. In less developed countries, contraceptive discontinuation due to health concerns is generally higher and these complaints are often related to service quality and reasons for such discontinuation remain a major concern for family planning program [6, 7].

In Ethiopia, according to Ethiopian Demographic Health survey (EDHS) in 2011, all modern contraceptive methods discontinuation rate was 37% and among them implant discontinuation rate within 12 months was just 5% and another study conducted in Tigray showed that the Implanon discontinuation rate in 1 year was 16% [8, 9]. Studies suggested that pre insertion counseling, education, age of the women and having no children are some of the predictors of early discontinuation [10, 11].

Few studies conducted on the magnitude of the early discontinuation rate of Implanon and its determinants among women’s of Implanon users in Ethiopia. The purpose of this study was to assess the magnitude of the early discontinuation rate of Implanon and to identify its determinants among women who ever used Implanon in 2016 in Dale district, Southern Ethiopia, 2017.

## Methods

### Study setting and populations

Community-based cross sectional study was conducted in Dale district, Southern Ethiopia, from January to February 2017. Among 4320 women for whom Implanon was inserted in the last 1 year, since January 01/2016 to December 31/2016 in Dale district, 711 participants were selected. Simple random sampling techniques were used to select study participants and were excluded women who ever inserted Implanon before January, 2016 & after December, 2016.

### Outcome variable

#### Discontinuation rate of Implanon

##### Independent variable

Socio-demographic factors, reproductive factors, psychological & medical factors, facing of side effects, service related & health facility factors and client & Partner factors were variables included in this study.

### Sample size determination

The sample size for this particular study was calculated using a formula for a single population proportion considering the following assumptions.

95% confidence level with margin of error (4%).

Proportion (P): proportion of the discontinuation rate of Implanon was 16% among women who.

ever used Implanon in Tigray [9].

Substituting in Kish Leslie (1996) single population proportion formula, gives: -.

$$ {\displaystyle \begin{array}{l}\mathrm{n}=\left(\mathrm{Z}\upalpha /2\right)2\mathrm{p}\ \left(1\hbox{-} \mathrm{p}\right)\\ {}\mathrm{d}2\\ {}\mathrm{N}=(1.96)2\ (0.16)\ (0.84)=(3.8416)\ (0.1344)=323\\ {}(0.04)2\ 0.0016\end{array}} $$

Where

N = required sample size

Z = critical value for normal distribution at 95% confidence level, which equals to 1.96 (z value atα =0.05, two tailed)

Expected proportion (P) = proportion to the discontinuation rate of Implanon was 16%.

Desired precision (d) = 0.04 (4% margin of error)

Design effect = 2

Sample size = 646

Contingency (for non-response =10%) =65

The final sample size = 711

The sample size for second objective was also calculated using double population proportion, 80% power, 95% CI using identified factors in previous study such as satisfaction, follow up and side effect. However, the calculated sample size of the first objective was higher. Thus the sample size calculated using single population proportion formula taken sufficient enough to measure the magnitude of early Implanon discontinuation & associated factors among women who ever used Implanon.

### Sampling procedure

Out of 33 kebeles (the smallest administration unit) of the study area 10 kebeles were selected using simple random sampling method. List of Implanon users were taken from the family planning, registration book of each selected kebele health post and health centers. Government owned health centers and health posts are supposed to serve nearly 90% of Implanon users [2, 5]. It is valid and complete. For the purpose of maintaining confidentiality and privacy issue an identification number was given by the researchers for each user in the place of their name and then that given number and address were used to select the study participants. A systematic random sampling method was used until the sample size for each kebeles was enough. Then study subjects from each kebeles were achieved through house to house interview. With the total amount of the Implanon users from selected 10 kebeles where *N* = 1416 the proportionally allocated sample size was in = 711 an interval of k = 1416/711 = 2 was used to determine the number of women who ever used Implanon for each kebeles. Women how were not at home during the visit, repeated households visit were done to decrease the non-response rate.

### Data collection tools &procedure

Ten diploma nurses as data collectors and two-degree holder nurse as supervisors were used. A structured interview based questionnaire was used to collect the data. It was developed by the investigators after reviewing different literature.

Questionnaire first prepared in English and translated to Sidama Afoo by an official translator and to check whether the translation was consistent with the English version. The questionnaire was back retranslated to English by another translator. The questionnaire was adapted from reviewing different literatures and scientific facts [12, 13]. The data were collected from selected kebeles and all women who ever used Implanon were interviewed house to house in a place where the participants felt free to express their feelings and ideas. Additionally, in occasions where the women have not accessed for absence, up to two attempts was endeavored for interview to lower the non-response rate.

Among the discontinuers, further information was asked the date of insertion, duration of Implanon used and the reason for removed during the data collection period.

### Data quality control

Ten diploma nurses of data collectors and two-degree holder nurses of supervisors were recruited and trained on the objectives of the study, data collection tools and interview techniques for 2 days by the investigators. The data collectors were selected from that reside in the study area and fluent in the local language. A questionnaire was pretested in 5% (17 women) outside of the study area, in Wonsho districts far from 8 km. Based on the pretest, necessary modification was made on the questions and the data of the pretest were excluded from the actual data analysis. The data collectors were filled and checked the completeness of each questioner before leaving the interviewee. Moreover, the supervisors also were checked on a daily basis for completeness and consistency of each questionnaire. Re interview of randomly selected study participants and compared against the previously filled questioners by the data collectors.

The collected data were cleaned, coded and entered into Epi- Info version 3.5.1 and then exported to Statistical Package for the Social Sciences (SPSS) version 20 windows program for further analysis.

### Operational definition

Discontinuation of Implanon: when a woman removed Implanon contraceptive method due to health concerns, accidental loss from inserted site and any reason done by a health worker within 1 years of use following insertion.

Health Concerns: previously existed physiological illness or medically known condition, which may result fear of the use of Implanon contraceptive methods.

Knowledge of contraception methods: a woman aware of at least one method of contraceptives.

Misconception: without scientific evidence, the women perceived Implanon contraceptive method can cause infertility, paralysis and shifting to other sites.

Partner involvement: husbands accompanies women in the health facility during Implanon insertion.

Prolonged menstrual bleeding: menstrual flow lasting more than 7 consecutive days Shifting/switching of contraceptive method: if the women need to change from one contraceptive to another contraceptive method if the woman needs and with medical advice to change.

Side effect: is any health problem related to the use of current contraceptive methods.

### Data processing and analysis

The descriptive statistics were used to measure central tendency, frequencies, cross tabulations, proportion and measure of variation to describe the study subjects and used to check for missed values. Bivariate and multiple logistic regression analysis were used to compute the effect of factor(s) on the outcome variables and to control possible confounds. Then all variables have a *p* value ≤0.25 in the bivariate analysis were further entered into multiple logistic regression model. The odds ratio was used to measure strengthen and identify factors associated with discontinuation rate of Implanon. In all analyses, *P* value < 0.05 was considered as a level of significance. In order to assess the goodness of fit of the final model Hosmer and Lemeshow goodness-of –fit test was applied. Finally, the results were presented using Adjusted Odds Ratio (AOR) and confidence level (95% Confidence Interval (CI)).

## Results

### Socio-demographic characteristics

A total of six hundred eighty-three (683) participants has responded to the questionnaires, making a response rate of 96.1%. The remaining 28 were unresponsive, so all the analysis was done on 683 women. The age of study participants was between < 20 and 35 + years with the mean age 24.5 ± 4.8 years. The majority of the participants 673 (98.5%) were married, 369 (54%) were not educated, 508 (74.4%) were Protestant by religion and 487 (71.3%) were self-dependent by occupation. More than three quarter of women from Implanon users (*n* = 683), 562 (82.3%) were Sidama by ethnicity. For more detail, please see Table 1.

**Table 1:** Socio-demographic status of women who ever used Implanon in 2016 in Dale district, Southern Ethiopia, 2017 (*n* = 683)

Variables	Frequency	Percent
Women’s age		
< 20	80	11.7
20–24	331	48.5
25–29	182	26.6
30–34	52	7.6
35+	38	5.6
Marital status		
Married	673	98.5
Others (single, divorced, widowed)	10	1.5
Religion		
Protestant	508	73.4
Muslims	119	17.4
Orthodox	56	8.2
Educational status		
No formal education	369	54
Primary	262	38.4
Secondary & above	52	7.6
Occupation		
Farmers	487	71.3
Others (Employed, student, merchant)	196	28.7
Ethnicity		
Sidama	562	82.3
Amahara	49	7.2
Wolayta	47	6.9
Others (Gurage, Silte)	25	3.7

### Reproductive history of study participants

Among 683 participants, 679 (99.4%) had live children with a mean (+ Standard Deviation (SD)) 2.82 ± 1.33 during data collection. With regard to abortion from Implanon users (*n* = 683), 32 (4.7%) had a history of abortion between one and three times with a mean (+SD) 1.44 ± 0.61 during data collection. For more detail, please see Table 2.

**Table 2:** Reproductive history and client characteristics of women who ever used Implanon in 2015/2016 in Dale district, Southern Ethiopia, 2017 (*n* = 683)

Variables	Frequency	Percent
Live children		
Yes	679	99.4
No	4	0.6
Number of children		
0	4	.6
1–3	480	70.27
4–5	178	26.1
6+	21	14.1
History of abortion		3.1
Yes	32	4.7
No	651	95.3
Number of abortions		
1	20	62.5
2	10	31.3
3	2	6.3

### Past contraceptive history & counseling status and place of insertion

Among all 411 (60.2%) ever heard about any contraception before Implanon insertion, 508 (74.4%) were counseled about contraception, of which 217 (42.7%) were counseled by the health care provider individually. For more detail, please see Table 3.

**Table 3:** Past contraceptive history before Implanon insertion & the counseling status of women who ever used Implanon within the last 1 year in Dale District, Southern Ethiopia, 2017

Variables	Frequency	Percent
Information on any contraception (*N* = 683)		
Before Implanon insertion		
Ever heard	411	60.2
Not ever heard	272	39.8
Ever heard about Implanon Before used (*N* = 411)		
Yes	242	58.9
No	169	41.1
Counseling about Implanon		
Yes	508	74.4
No	175	25.6
Types of counseling (*N* = 508)		
Individual counseling	217	42.7
Mass counseling	208	41
With husband counseling	83	16.3
Information during counseling(*N* > 683)		
Effectiveness	217	31.8
Duration of action	204	29.9
Benefit	181	26.5
Side effects	152	22.3

More than half of participants 430 (63%) were inserted the Implanon in health centers, and 216 (31.6%) were in health posts.

### Magnitude of Implanon discontinuation rate

**Fig. 1:**
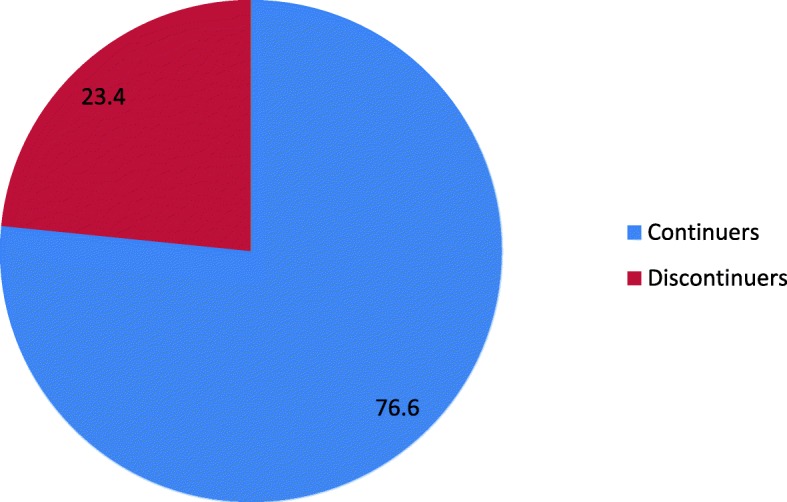
Number of women who ever used Implanon in the last 1 year in Dale district, Southern Ethiopia, 2017

Out of the 683 women who ever used Implanon during the last 1 year, of these 160 (23.4%) women discontinued. Women who were removed the Implanon were used the Implanonfor the duration of between 4 and 12monts with a mean of 9.6 ± 2.5 months. Among over all discontinued 35 (5.12%) of the Implanon discontinues had removed between 4 and 6 months following insertion and 125 (18.3%) of discontinues had removed 7–12 months. For more, please see Fig. 1.

**Fig. 2:**
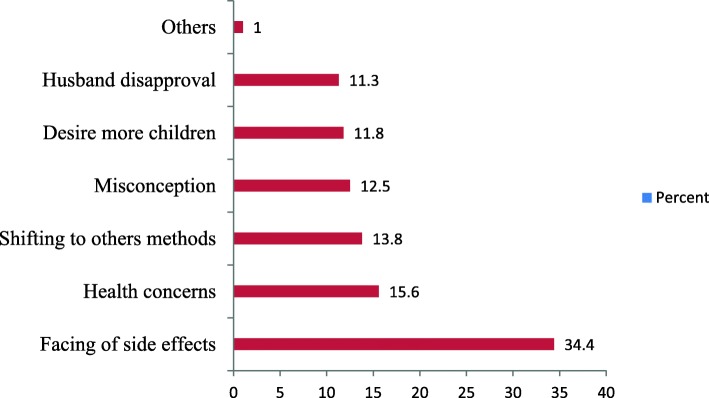
The reasons for Implanon discontinuation among women who ever used Implanon in 2016 in Dale District, Southern Ethiopia, 2017

The main reasons for discontinuation of Implanon were 55 (34.4%) facing of side effects, 25 (15.6%) health concerns, 22 (13.8%) shifting to other methods, 20 (12.5%) misconception 19 (11.8%) desire to have more children &18 (11.3%) husband’s disapproval. Distance of health facility for community and religion concern was also other less frequent reasons for Implanon discontinuation. See Fig. 2.

The most common complaints among facing of side effects were 69 (52.7%) irregular menses, 20 (15.3%) weight gain & 11 (8.4%) dizziness. For more, please see Fig. 2. In general, after removal of Implanon contraceptive method, 160 women who were removed Implanon 22 (13.75%) have shifted another method, of these 13 (59%) was used Depo-Provera, 5 (22.7%) were used Intra Uterine Contraceptive Devise (IUCD) and 4 (18.2%) were used Pills.

Among 160 women who were removed Implanon 138 (86.25%) were stopped Implanon contraceptive. Of these, 44 (27.5%) women didn’t use any contraceptive methods for the reasons of medical indication, 94 (58.7%) women will be ready to conceive a pregnancy.

### Factors associated with discontinuation of Implanon

The results demonstrated that as the age of the women increases less likely the women to discontinue the methods. Women age 20–24 years, 48%, 25–29 years, 56%, 35+ years, 92% less likely to discontinue the methods compared to women less than 20 years (AOR =. 44 (95% CI: 23-. 85), (AOR =. 52 (95% CI: 27-. 96), (AOR =. 08 (95% CI: 02-. 41) respectively. Women who earn a monthly income of greater than 1000 Eth birr, 55% less likely to discontinue the methods compared to those women who earn less than 500 Eth birr=. 45 (95% CI: 20-. 97). Women who do not have information on contraceptive methods before Implanon insertion were 1.52 times more likely to discontinue Implanon as compared with those who have information on contraceptive methods (AOR = 1.52 (95% CI: 1.1–2.28). Those who weren’t appointed to follow up were 3.13 times more likely to discontinue Implanon as compared with those who have followed up after insertion (AOR = 3.13 (95% CI: 2.0–4.95). Women who weren’t counseled about the side effects during Implanon insertion were 1.93 times more likely to discontinue Implanon as compared to those who counseled (AOR = 1.93 (95% CI: 1.27–2.93). Women who weren’t satisfy by the service given during the insertion of Implanon were 2.55 times more likely to discontinue Implanon as compared with those who have satisfied during Implanon insertion (AOR = 2.55 (95% CI: 1.63–3.97) and women who were not choose the method by themselves were 1,83 times more likely to have discontinuation of Implanon (AOR = 1.83 (95% CI: 1.18–2.83) as compared with those who choose the method themselves. For more, please see Table 4.

**Table 4:** Factors associated with discontinuation rate of Implanon among women who ever used Implanon in 2015/2016 in Dale District, Southern Ethiopia, 2017

Variables	Discontinuation Rate		Crude OR 95% CI	Adjusted OR 95% CI
	Yes (%)	No (%)		
Age of the women				
< 20	35(43.8)	45(56.3)	1	1
20–24	71(21.5)	260(78.5)	.35(.21–.58)**	.52(. 27–.96)*
25–29	37(20.3)	145(79.7)	.33(.18–.58)**	.44(.23–.85)*
30–34	14(26.9)	38(73.1)	.47(.22–1)*	.73(.31–1.7)
35+	2(5.3)	36(94.7)	.07(.01–.31)**	.08(0.2–.41)**
Monthly income				
< 500 Eth Birr	247(74.6)	84(25.4)	1	1
500–1000 Eth Birr	218(77)	65(23)	.87(.60–1.27)	.8(.53–1.2)
> 1000 Eth Birr	59(85.5)	10(14.5)	.5(.24–1.0)*	.45(.20–.97)*
Live children				
Yes	522(76.9%)	157(23.1%)	1	1
No	2(50%)	2(50%)	3.32(0.46–23.79)	1.42(0.55–3.64)
Information on FP				
Yes	336(81.8%)	75(18.2%)	1	1
No	188(69.1%)	84(30.9%)	2.0(1.39–2.86)*	1.52(1.1–2.28)*
Counseling				
Yes	409(80.5%)	99(19.5%)	1	1
No	115(65.7%)	60(34.3%)	2.15(1.47–3.15)**	1.93(1.27–2.93)**
Choice				
Yes	272(83.7%)	5 3(16.3%)	1	1
No	252(70.4%)	106(29.6%)	2.15(1.48–3.13)*	1.83(1.18–2.83)**
Facing of side effect				
Yes	70(53.4%)	61(46.6%)	1	1
No	454(82.2%)	98(17.8%)	4.03(2.68–6.06)*	1.54(0.78–3.04)
Satisfaction				
Yes	261(88.2%)	35(11.8%)	1	1
No	263(68%)	124(32%)	3.51(2.32–5.31)**	2.55(1.63–3.97)**
Follow up				
Yes	227(88.3%)	30(11.7%)	1	1
No	297(69.7)	129(30.3%)	3.28(2.13–5.06)*	3.13(2.0–4.95)**
FP previous used				
Yes	225(79.2%)	67(20.8%)	1	1
No	299(76.5%)	92(23.5%)	1.30(0.91–1.86)	0.99(0.66–1.48)

NB = **p < 0.001; *p < 0.05; Eth birr (Ethiopian birr)

## Discussion

This study found that a higher number of Implanon users discontinued early. According to the multivariate regression analysis, age, monthly income, counseling, information on family planning who chooses the methods by themselves and follow up were identified factors for early discontinuation. The study also found the majority of the users were younger’s. This finding is similar to a study done in Tigray [13]. This might be youngers initially prefer the methods because it doesn’t require to visit health facilities every time until they need to remove it. Thus the users might think that Implanon method is easy to keep their privacy compared to other methods like injection and pills. Other reasons might need further research also important to dig out in detail.

The discontinuation rate in this study was substantially higher compared to similar study conducted in Tigray in Northern Ethiopia and also Central Nigeria, Northern Nigeria, India and Brazil [9, 14–17] and this could be due to the difference in educational status of the study participant’s, as majority of women in the study conducted in Northern Nigeria, Central Nigeria and India were secondary and higher level [14–16]. The other possible reason might also be due to age difference, because women of the current study were younger age than the previous studies [14–16] as result of desire to have children and lack of tolerance for the side effects. It might be due to study setting as the current study was conducted in rural whereas the others were either in urban or in both urban and rural. The final reason might be due to inadequate pre insertion counseling, particularly about the expected side effects of the method.

This study finding also a bit lower than other study conducted in the Arsi zone in South East Ethiopia, United Kingdom and Australia in which showed that around 25–30.2% of Implanon users had discontinued and this might be due to the difference in study design and context [18–20].

This study revealed that as the age of the women increases less likely to discontinue the Implanon compared to those who were at younger than 20 years. This might be while the women getting older, they demand to have a child decreases because they might have enough number of children they want and also the level of tolerance of the side effect increases. This finding also in line with the study done in southern Ethiopia [10].

Women who have relatively high monthly income less likely to discontinue the methods compared to those who earn less. This might be in connection with their level of educational status, those who earn more is better educated than their counter. Education is helpful to understand and outweigh the advantage and disadvantage of the methods they are using. This finding also in line with the studies done in southern Ethiopia, upper Egypt and Nigeria [10, 21, 22].

Women who don’t have pre- insertion counseling and lack of information on family planning were 1.93 and 1.52 times more likely to discontinue as compared to those who were counseled and have information. Lack of proper counseling and information regarding the side effects, method change were more likely to result in a negative attitude towards methods whenever they experience the side effects. The possible explanation could be if women have very clear information about the provided service, they will give up with minor side effects and may have a higher chance to continue the method. In addition, the family planning counselor should review a woman reproductive plan (delayer, spacer, and limiter) with her before providing the service. So that women can choose the appropriate method for their fertility plan by their own as per the standard informed choice [23]. This finding is also in line with studies done in Debre Markos town, northwest Ethiopia and Louis City [24, 25].

Women who didn’t satisfy by the service given during the insertion of Implanon were 2.55 times more likely to discontinue Implanon as compared to those who satisfied. This is because women who were not satisfied with their choice, privacy and explanation of the service provider about the importance of using Implanon during insertion may discontinue the method earlier. This finding is similar with study conducted in Tigray in Northern Ethiopia [13].

Women who didn’t choose the method by themselves were 1.83 times more likely to discontinue Implanon as compared to those who choose. This might be those who choose the methods by themselves have information about the method and expected side effects. This finding also in line with studies done in low income countries [26].

Lastly, women who didn’t appoint for follow up were 3.13 times more likely to discontinue the Implanon as compared to those who had followed up. This might be during follow up visit, if any compliant coming from the client, they will get appropriate solution from the health workers and also there might be post insertion counseling on the expected side effects specific to Implanon. This finding is also similar to studies done in Tigray in Northern Ethiopia and Louis City [9, 13, 25].

## Limitation

This study finding might not be used to generalize for women in urban places and recall bias can be introduced according to date of insertion and removal of Implanon.

## Conclusions

Implanon discontinuation rate in this study was substantially high and the main factors for discontinuation were lack of pre insertion counseling, lack of satisfaction due to poor service delivery and lack of appointment for follow up. Thus, proper pre-insertion counseling and follow up will improve the continuation rate of Implanon.

## Abbreviations

AOR: Adjusted odds ratio
CI: Confidence interval
EDHS: Ethiopian demographic health survey
IUCD: Intra uterine contraceptives
SD: Standard deviation
SPSS: Statistical package for the social sciences
